# Retinal Findings and Cardiovascular Risk: Prognostic Conditions, Novel Biomarkers, and Emerging Image Analysis Techniques

**DOI:** 10.3390/jpm13111564

**Published:** 2023-10-31

**Authors:** Joseph Colcombe, Rusdeep Mundae, Alexis Kaiser, Jacques Bijon, Yasha Modi

**Affiliations:** 1 Department of Ophthalmology, NYU Langone Medical Center, New York, NY 10016, USA; joseph.colcombe@nyulangone.org (J.C.); rusdeep.mundae@nyulangone.org (R.M.); 2 College of Arts and Sciences, University of Pennsylvania, Philadelphia, PA 19104, USA; 3 Vitreous Retina Macula Consultants of New York, New York, NY 10022, USA; jacques.bijon@nyulangone.org

**Keywords:** cardiovascular risk, stroke, myocardial infarction, retinal vascular occlusions, subretinal drusenoid deposits, retinal ischemic perivascular lesions, optical coherence tomography, optical coherence tomography angiography

## Abstract

Many retinal diseases and imaging findings have pathophysiologic underpinnings in the function of the cardiovascular system. Myriad retinal conditions, new imaging biomarkers, and novel image analysis techniques have been investigated for their association with future cardiovascular risk or utility in cardiovascular risk prognostication. An intensive literature search was performed to identify relevant articles indexed in PubMed, Scopus, and Google Scholar for a targeted narrative review. This review investigates the literature on specific retinal disease states, such as retinal arterial and venous occlusions and cotton wool spots, that portend significantly increased risk of future cardiovascular events, such as stroke or myocardial infarction, and the implications for personalized patient counseling. Furthermore, conditions diagnosed primarily through retinal bioimaging, such as paracentral acute middle maculopathy and the newly discovered entity known as a retinal ischemic perivascular lesion, may be associated with future incident cardiovascular morbidity and are also discussed. As ever-more-sophisticated imaging biomarkers and analysis techniques are developed, the review concludes with a focused analysis of optical coherence tomography and optical coherence tomography angiography biomarkers under investigation for potential value in prognostication and personalized therapy in cardiovascular disease.

## 1. Introduction

The retina is an intricate structure tightly interwoven with the body’s cardiovascular system. It is one of the most metabolically demanding organs and is a major consumer of oxygen on a per-weight basis [1]. A complex network of vessels supplies the different layers of the retina. The inner two-thirds is supplied by capillary networks from the central retinal artery. The superficial vascular plexus resides in the ganglion cell layer; the intermediate and deep vascular plexuses are superior and inferior to the inner nuclear layer (collectively, the deep capillary complex). Additionally, the radial peripapillary capillaries (from the posterior ciliary arteries) run parallel to the retinal nerve fiber layer [2]. The outer one-third of the retina, beginning with the outer plexiform layer, is supplied indirectly by the choroidal blood supply (the choriocapillaris).

Underlying vascular or cardiac dysfunction is therefore frequently reflected in the health of the retina due to its profound vascularity. In an age of increasingly personalized medicine, optimal clinical counseling and disease management and surveillance require targeted prognostication and risk estimation from a variety of data. This article will explore a handful of discrete retinal conditions that either confer increased cardiovascular risk (central retinal artery occlusion, central vein occlusion, cotton wool spots, and subretinal drusenoid deposits) or do not conclusively increase CVD odds (acute macular neuroretinopathy), despite the theorized pathophysiologic mechanism and implications in individualized patient therapy. Attention will then be turned to retinal imaging findings potentially associated with cardiovascular disease (paracentral acute middle maculopathy and the newly described retinal ischemic perivascular lesion). Lastly, a review of exploratory research utilizing novel qualitative and quantitative optical coherence tomography (OCT) and optical coherence tomography angiography (OCTA) parameters that may carry cardiovascular prognostic value will be presented, with a discussion of the implications in personalized medicine.

## 2. Methods

A literature search was conducted in PubMed, Scopus, and Google Scholar using English keywords such as: “retina AND cardiovascular risk”, “retina AND myocardial infarction”, “retina AND stroke”, “CRAO”, “CRVO”, “cotton wool spots”, “AMD AND cardiovascular risk”, “subretinal drusenoid deposits AND cardiovascular risk”, “PAMM”, “AMN”, “RIPL” “fundus photos cardiovascular”, “OCT retina cardiovascular”, “OCTA retina cardiovascular”, and associated topics and combinations of the aforementioned words. Articles published at any date were considered. Major inclusion criteria included English language or accompanying translation into English with publication. Non-English language articles without an accompanying translation were excluded. Articles were selected for incorporation into this narrative review based on the strength and uniqueness of their evidence for original contributions or clarity and comprehensiveness for reviews. When feasible, original research articles were prioritized over review articles.

## 3. Findings

### 3.1. Retinal Conditions Established in the Literature as Portending Increased Cardiovascular Risk

#### 3.1.1. Central Retinal Artery Occlusion

Central retinal artery occlusion (CRAO) is caused by a blockage in the blood flow of the central retinal artery and commonly presents with rapid, painless, monocular vision loss and a relative afferent pupillary defect. Retinal whitening with sparing of the foveal center (cherry red spot) is seen on fundoscopy. Optical coherence tomography (OCT) imaging of the macula demonstrates a thickened and hyperreflective inner retina in the acute setting. CRAO is relatively uncommon. Age- and sex-adjusted incidence is approximately 1.8–1.9 per 100,000 person-years in America; this incidence increases to approximately 10 per 100,000 person-years over the age of 80 [3]. At least 95% of CRAO events have a thromboembolic origin; in 5% of cases or less, the etiology is arteritic in the setting of giant cell arteritis [4]. CRAO that occurs due to arterial thromboembolism is considered mechanistically equivalent to an embolic ischemic stroke, and as such, CRAO is officially classified as a form of acute ischemic stroke by the AHA Stroke Council [5].

There exists substantial evidence that CRAO confers significant cardiovascular risk to patients. Patients with CRAO have a high burden of concurrent carotid pathology, such as critical atherosclerosis or dissection. One study identified major carotid disease in 36% of CRAO patients [6]. Cardiovascular workup as an inpatient on CRAO patients can also uncover significant additional morbidity. In the prior study, 20% of CRAO patients had a profoundly abnormal echocardiography result (such as substantial valvular disease or heart failure). Importantly, patients are at an increased risk of stroke, MI, and death after CRAO. In the first year after an event of CRAO, a patient has over a 25% chance of suffering an acute stroke, MI, or dying—over 4 times higher than matched patients who suffered a TIA [6]. The incidence of acute stroke especially is most pronounced in the first week after diagnosis, when CRAO patients have a 44 times higher incidence of acute stroke compared to the ensuing weeks [7].

CRAO is intricately connected to myocardial infarction risk as well, and CRAO frequently occurs after and before MI [8]. Hospitalized CRAO patients have a significantly elevated risk of acute MI while inpatient, perhaps reflecting the advanced systemic cardiovascular disease present in most sufferers of retinal artery occlusions. Although no direct comparisons can be made, patients hospitalized with CRAO in America appear to have a risk of acute in-hospital myocardial infarction on the order of 3.7%. For comparison, large surveys of all patients admitted at Veterans Affairs hospitals have established baseline acute MI risk on the order of less than 0.5% during an inpatient stay [9,10]. Interestingly, retinal ischemic damage due to retinal vascular occlusions in mice (simulating reperfusion after an arterial occlusion) has been experimentally linked to CD4+ T-cell-mediated cytokine and inflammatory responses [11]. CD4+ T cells also exhibit a characteristic biphasic activation during acute MI (reaching maximal activity in myocardial tissue 3 days after infarction) and heart failure (with a large spike in T-cell transmigration 20 times normal levels during congestive heart failure) [12]. CD4+ T cells and the associated pro-inflammatory signaling milieu they promote during ischemic damage may serve as a cellular biomarker for reperfusion injury after ischemia in the retina and heart. The role of T cells in heart disease is an area of active research [13,14,15], and future studies are warranted to evaluate the role of immune cells in retinal conditions linked to systemic cardiovascular compromise.

CRAO is additionally associated with an increased risk of arrhythmia, especially atrial fibrillation. CRAO patients are more than 12 times as likely as the general population to have atrial fibrillation. Compared to health-, sex-, and age-matched controls, a diagnosis of CRAO also increases the risk of developing atrial fibrillation in the two years following diagnosis by over 60% [16,17]. Overall, CRAO is known to drastically increase subsequent stroke and myocardial infarction, and carries strong associations with other systemic cardiovascular diseases, including arrhythmia (especially atrial fibrillation).

A diagnosis of acute CRAO should result in immediate emergency department referral, ideally at a stroke center. According to the latest preferred practice pattern guidelines of the American Academy of Ophthalmology, within the first 24 h, an extensive workup should be completed on an inpatient basis. This includes thorough labwork such as a comprehensive metabolic panel, complete blood count, lipid panel, hemoglobin A1C, erythrocyte sedimentation rate, C-reactive protein, and coagulation profile (prothrombin time and partial thromboplastin time). Cardiac evaluation such as electrocardiogram and echocardiography should be performed, and neuroimaging of the brain is also indicated (ideally, noncontrast magnetic resonance imaging of the brain) and vessels (computed tomography angiography of the head and neck, magnetic resonance angiography of the head and neck, or bilateral carotid ultrasounds) [18]. Findings suggestive of chronic CRAO should prompt physicians to refer or schedule many of the above procedures in an ambulatory setting, as new cardiovascular disease (such as atrial fibrillation or critical carotid atherosclerosis) may be unmasked, and systemic intervention (such as anticoagulation) may be warranted.

The active treatment of CRAO in the acute setting, with the goal of renewing or augmenting retinal perfusion, is controversial. Therapies such as hyperbaric oxygen, ocular massage, anterior chamber paracentesis, intravenous mannitol or acetazolamide, and corticosteroids have all been utilized, but none have definitively been shown to improve outcomes [19]. As mentioned previously, CRAO is now recognized as a specific type of ischemic stroke by major neurovascular and cardiovascular guideline organizations. Therefore, tissue plasminogen activator (tPA) (intravenously, or, in select centers, directly into the ophthalmic artery via cannulation of the femoral artery) is administered in a significant number of academic health centers in America within the first few hours of visual symptoms [20]. Although this approach is not standard, and carries profound risk, a growing body of evidence supports an improvement in visual acuity with early administration of tPA [21,22]. However, due to the heterogenous nature of CRAO protocols at different institutions and lack of consensus, most studies evaluating the use of tPA in CRAO do not contain a control arm, and the definitive net benefit of tPA for CRAO has not been conclusively demonstrated [23].

Therefore, conclusive management of acute CRAO centers on diligent investigation in the inpatient setting for systemic cardiovascular and cerebrovascular comorbid disease and temporal arteritis. This requires an array of blood chemistry, labwork, and cardiovascular and neurologic imaging. The best treatment practice for CRAO to restore vision is equivocal, but tPA is increasingly used in select candidates.

#### 3.1.2. Central Retinal Vein Occlusion

**Figure 1:**
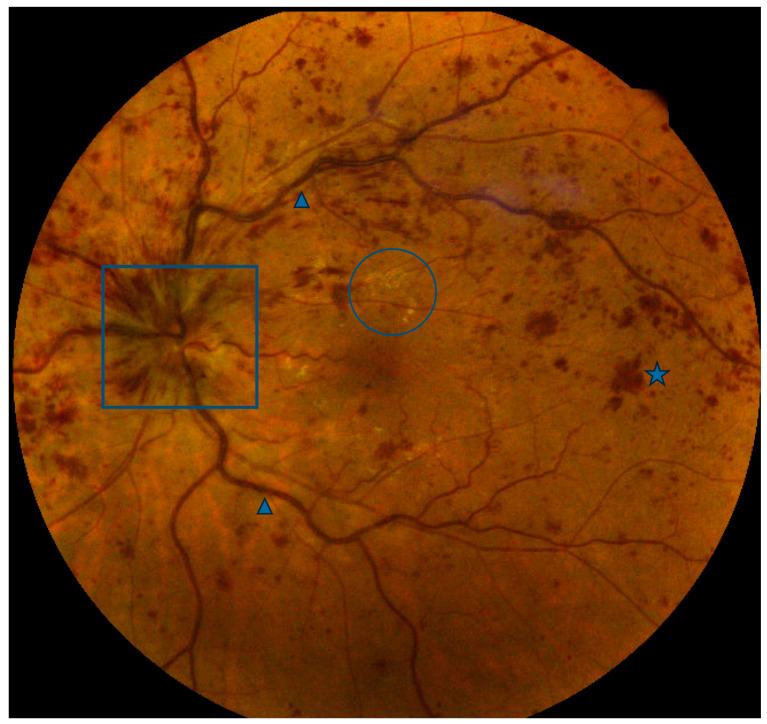
Color fundus photo of a central retinal vein occlusion with optic disc edema (box), venous dilation and tortuosity (arrowheads), intraretinal hemorrhages (one of many, star), and exudates (one group of many, circle).

Central retinal vein occlusions are produced by blockage of the central retinal vein at or posterior to the optic nerve head. The occlusion results in increased retinal vein and capillary pressure and consequent hemorrhage with possible macular edema. A “blood and thunder” fundus is classic, but the findings may be subtle in less severe forms (Figure 1). In the Beaver Dam Eye Study, CRVOs were found to have approximately a 0.5% cumulative incidence, with branch retinal vein occlusions (BRVOs) at an incidence of 1.8% [24]. Although CRVO can be triggered by any state that causes a hypercoagulable local environment (such as by select blood dyscrasias including polycythemia vera, multiple myeloma, Waldenstrom macroglobulinemia, or clotting disorders like factor V Leiden disease, antiphospholipid syndrome, and mutations in antithrombin III and protein S or protein C), the exact pathogenesis remains elusive. Retinal vein occlusions are most commonly conjectured to occur secondary to vascular wall changes. As a shared adventitial layer covers both retinal arteries and veins, arteriosclerosis affecting the adjacent arterial vessels causes wall thickening, which compresses and damages the neighboring venous wall [25]. Endothelial damage and inelasticity also result, with ensuing venous stasis creating a conducive environment for thrombosis. In the case of CRVO, the compression of the central retinal vein by the central retinal artery most likely occurs at the level of the lamina cribrosa. Unsurprisingly, processes that are associated with increased pressure at the lamina cribrosa (i.e., glaucoma) increase the risk of retinal vein occlusion [26].

Due to this theorized mechanism, CRVO has been examined for its relationship to systemic cardiovascular disease. Hypertension, diabetes, and open-angle glaucoma all are risk factors for CRVO [27]. CRVO itself appears to increase the risk of incident cardiovascular disease by over 10% compared to an age-matched cohort [28]. Patients with any form of retinal vein occlusion have higher calculated cardiovascular risk (cCVDR) scores as determined using the Framingham algorithm. In one study, the 10-year cCVDR for RVO patients was approximately 5% higher (20.6% versus 15.7%) than age-matched controls [29]. RVO also appears to increase the risk of myocardial infarction. A propensity score-matched and -adjusted study found that retinal vein occlusion patients had a modestly higher risk of acute MI (hazard ratio of 1.21; 95% CI: 1.13 to 1.30) compared to age-, sex-, and non-cardiovascular-comorbidity-matched control patients. Moreover, among all retinal vein occlusion patients, CRVO patients had a significantly higher occurrence rate of acute myocardial infarction than BRVO patients (3.6% versus 3.12%, (*p* < 0.0001)) [30]. Indeed, RVOs as a whole increase the risk of heart myocardial infarction and peripheral arterial disease by approximately 25% and increase the risk of congestive heart failure by approximately 50% in a recent cohort study meta-analysis [31].

The increased cardiovascular risk conferred by retinal vein occlusions translates to cerebrovascular disease as well. A study by Bakhoum et al., which included the records of over 80,000 individuals, found that RVO significantly increased the risk of stroke, even after adjusting for age, sex, and known cardiovascular disease and other risk factors. RVO patients had an odds ratio of 1.73 for stroke occurrence, and this association was notably stronger in younger individuals (<50), where the odds ratio was found to be 3.06 (*p* < 0.001) [32]. A prior age- and sex-matched study looking at over 1 million randomly selected Korean residents also found an increased risk of stroke in RVO patients (16.8% versus 10.7%, HR 1.48), and the hazard ratio was also greater for younger patients aged <50 years (2.69). Conflictingly, a meta-analysis published by the *Journal of the American Heart Association* found that RVO was associated with a greater incidence of stroke (RR 1.5), even after controlling for established cardiovascular disease, but did not find that RVO was associated with stroke in patients under 50 years of age (*p* value 0.23) [33].

Contemporary research also suggests that personalized treatment of patients with RVO to decrease future CVD risk may be feasible. As retinal vein occlusions are both a risk factor for future cardiovascular events and more common in those with cardiovascular comorbidities, many patients with RVOs are on statin therapies for known cardiovascular risk reduction. A recent population-based case–control study centered on the Korean nationwide health database found that, in patients with newly diagnosed RVO, a significantly lower risk of cardiovascular events (stroke and MI) was found in RVO patients receiving statin therapy (adjusted OR 0.6040). A longer course of statin treatment was associated with an overall lower risk [34]. Although further research is necessary, these findings suggest that the occurrence of RVO may be an important dataset in individualized patient care. Close monitoring and cardiovascular workup (especially lipid profile, blood pressure screening, and echocardiography) may be reasonable. After a discussion with a cardiologist based on risk, statin therapy may be considered. Future research is required before uniformly providing statin therapy to patients with CRVO.

#### 3.1.3. Cotton Wool Spots

Cotton wool spots (CWS) are discrete pale retinal lesions that are caused by nerve fiber layer infarcts secondary to retinal arteriole occlusion. They are asymptomatic on their own, but commonly coexist with other ocular and systemic vascular disorders. CWS are common in patients with poorly treated diabetes mellitus and systemic hypertension and are transient findings on exam. CWS most frequently resolve by 3 months after they first appear, though they can last longer, especially in diabetic patients [35]. They leave behind a legacy of focal inner retinal thinning seen on macular OCT imaging and can result in long-term blind spots picked up on microperimetry and visual field testing [36].

CWS have long been investigated for their relationship to systemic cardiovascular disease. Indeed, cotton wool spots and other markers of hypertensive retinopathy such as arteriovenous nicking have been incorporated into one of the longest-running cardiovascular studies, the Atherosclerosis Risk in Communities (ARIC) study, which began in 1987. According to data from ARIC, CWS and other signs of hypertensive retinopathy confer a two- to fourfold increase in stroke risk over the ensuing 3 years after they are initially identified on exam [37]. Interestingly, cotton wool spots (along with retinal hemorrhages) may be more closely associated with intracerebral hemorrhagic strokes versus ischemic lacunar strokes than other stigmata of hypertensive retinopathy [38]. Furthermore, even after controlling for preexisting heart disease, hypertension, diabetes mellitus, and other cardiovascular risk factors, CWS (along with intraretinal hemorrhages and microaneurysms) are associated with a twofold higher risk of incident congestive heart failure diagnosis, and a threefold higher risk of acute events related to heart failure [39]. However, cotton wool spots, along with microaneurysms, focal arteriolar narrowing, and retinal hemorrhage, may reflect acute derangements in blood pressure and hypertensive damage at the time of exam. Some other signs of hypertensive retinopathy, especially arteriovenous nicking and generalized (not focal) arteriolar narrowing, appear to reflect more accurately the cumulative (i.e., years-long) retinal damage that accrues secondary to hypertension [40].

In sum, CWS are common among the constellation of findings seen in hypertensive retinopathy and diabetes, and their presence predicts increased risk of stroke- and heart-failure-related adverse events. Focused management of patients with CWS should entail blood pressure monitoring and antihypertensive treatment, along with diabetic evaluation (i.e., hemoglobin A1C) and appropriate lifestyle and medical management.

#### 3.1.4. Age-Related Macular Degeneration: Subretinal Drusenoid Deposits

**Figure 2:**
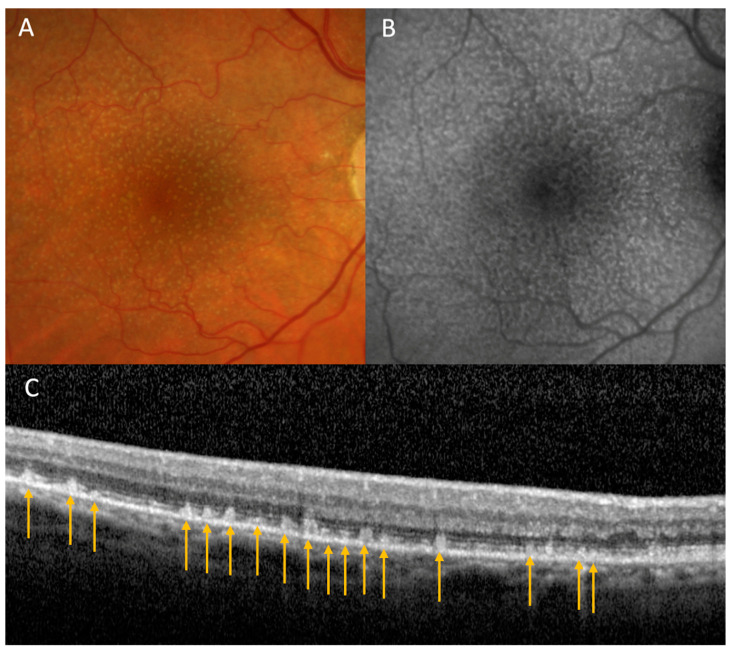
(**A**) Fundus photography demonstrating reticular pseudodrusen/subretinal drusenoid deposits in a dot pattern. (**B**) Fundus autofluoresence imaging with pinpoint hypoautofluorescent lesions with surrounding hyperautofluoresence. (**C**) Ocular coherence tomography imaging with hyperreflective deposits between the RPE and ellipsoid zone with some lesions puncturing through the ellipsoid zone into the outer retina, consistent with subretinal drusenoid deposits (arrows).

Age-related macular degeneration (AMD), a leading cause of irreversible vision loss in patients over the age of 50, is under active scrutiny for potential systemic health repercussions due to its ubiquity. The complex disorder has a prevalence of over 12% in American adults aged 40 or older, and the global burden of the disease may approach nearly 300 million people by 2040 [41,42]. Patients with AMD classically have drusen, yellow-lipid-heavy spheroid deposits, located between the retinal pigment epithelium (RPE) and Bruch’s membrane (BM). However, there exists a different subset of patients with AMD who have subretinal drusenoid deposits (SDD, also called reticular pseudodrusen), which are localized above the RPE and below the retina, often in a netlike or reticular arrangement (Figure 2). SDDs are thought to contain a different assortment of lipids than classical drusen [43,44].

The relationship of AMD with classical drusen and cardiovascular disease is controversial, and the evidence is questionable. Some studies have found an increased risk of cardiovascular disease in AMD [45,46,47]. However, no risk [48], or even decreased risk, has also been found [49,50]. For example, a recent study published in the *Journal of the American Heart Association* examined over 3,000,000 Korean patients to assess if AMD with visual disability (best corrected visual acuity of 20/100 or worse) was associated with increased incident cardiovascular disease. After nearly 10 years of follow-up, the study found that AMD with visual disability (especially in the setting of concurrent cardiac comorbidities) was significantly associated with an 18% higher risk of myocardial infarction, a 20% increase in ischemic stroke, and a 17% increase in cardiovascular disease [51]. However, classic AMD without visual disability was not associated with an increased risk of CVD. Although the relationship of classic AMD and cardiovascular risk is an area of active research, at present there is no consensus on this matter, and no convincing and reproducible body of literature suggests that classic AMD should trigger heightened suspicion for comorbid cardiovascular processes.

However, SDD do appear to potentially portend increased cardiovascular disease. A recent prospective study suggests that SDD confer substantial increased cardiovascular risk. Smith et al. examined 126 AMD patients: 62 with SDD and 64 without SDD (only classical drusen) over approximately 2 years. SDD were found to be significantly associated with lower high-density lipoprotein, cardiovascular disease, stroke, and the ARMS2 allele compared to AMD patients with drusen only. In fact, of the 51 patients who developed cardiovascular disease and stroke, 34 (66.7%) had AMD with SDD [52]. Previous studies have found that coronary artery disease significantly increases the risk (by over a factor of 2) of finding SDD lesions on exam. CAD confers no additional risk of finding classical drusen, further supporting a systemic relationship between cardiovascular disease and SDD [53]. Indeed, another recent study found an overwhelming association between AMD with SDD and cardiovascular disease: 42/43 (97.7%) SDD patients were found to have cardiovascular disease (heart pump compromise, valvular dysfunction, or carotid disease) versus 5/42 (11.9%) AMD patients without SDD. SDD was also again found to be associated with lower HDL [54]. Even impaired renal function, as measured by glomerular filtration rate, has been associated with the presence of SDD [55]. These discoveries, though exciting, are limited by small sample sizes and drawn from largely homogenous sample groups. SDDs may indeed confer increased CVD risk, but larger studies from more varied cohorts of patients are needed.

Overall, these findings should heighten clinician suspicion for cardiovascular disease in AMD patients. SDD especially should prompt consideration for systemic cardiovascular workup including lipid profile and hemoglobin A1C. In patients with SDDs and a family history of cardiovascular disease or any other systemic comorbidities such as hypertension, electrocardiography and echocardiography may also be reasonable to pursue due to the burgeoning association of SDD and various forms of systemic cardiovascular disease.

### 3.2. A Retinal Condition of Uncertain Systemic Cardiovascular Significance

#### Acute Macular Neuroretinopathy

**Figure 3:**
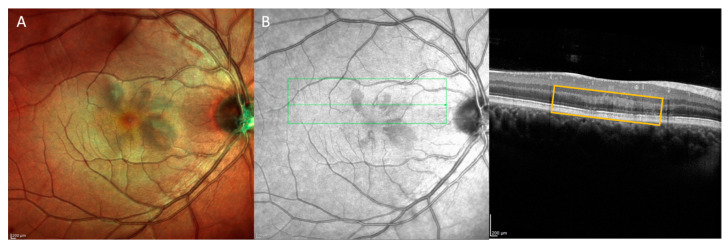
(**A**) Multicolor image demonstrating multiple wedge-shaped lesions pointed towards the fovea consistent with acute macular neuroretinopathy. (**B**) Ocular coherence tomography through several lesions demonstrating hyperreflectivity in the outer plexiform layer with ellipsoid zone hyporeflectivity (box).

Acute macular neuroretinopathy (AMN) presents as acute vision loss due to a paracentral scotoma (unilateral or bilateral), often in young patients, with characteristic brown or grey wedge-shaped lesions on the posterior pole pointed toward the fovea (Figure 3A). AMN is thought to be caused by ischemia or localized inflammation of the deep capillary plexus of the retina [56]. Unlike paracentral acute middle maculopathy, an OCT diagnosis to be discussed later in this review, significant outer retinal abnormalities are seen on OCT in patients with AMN. In AMN, the outer plexiform layer and outer nuclear layers are hyperreflective, and the ellipsoid zone appears disorganized and hyporeflective (Figure 3B) [57]. AMN is likely understudied in the current literature, perhaps due to the diagnostic challenges involved in making the diagnosis. The lesions, while difficult to see clinically, may be better visualized on the near-infrared en face imaging that accompanies the cross-sectional OCT. Nonetheless, given the diagnostic difficulties and potential pathophysiologic overlap with PAMM, determination of potential systemic cardiovascular associations attributed with AMN is difficult.

No study to date has examined the association between AMN occurrence and future cardiovascular risk; the majority of topical articles on the disease comprise case reports or small case series. AMN has been reported after pathology or circumstances as varied as recent mild viral illness, SARS-CoV-2 infection and vaccination, dengue fever, the Valsalva maneuver, leukemia, sympathomimetic drugs, caffeine use, and oral contraceptives, among many other conditions [58,59]. The most comprehensive review published to date on the disease, by Bhavsar et al. in 2016, examined 101 cases of AMN involving 156 eyes and found that over 80% of patients were female. Patients also were overwhelmingly young: the median age of onset was 29.5 years. Notably, recent infection or febrile illness was seen in nearly half (47.5%) of patients, and over one-third (35.6%) were prescribed oral contraceptives (though this may be a function of the heavily skewed female distribution of patients) [60]. Microvascular ischemia of the deep capillary plexus could feasibly be induced by many of the antecedent triggers noted in the literature, such as viral illness, oral contraceptives, shock, epinephrine, ephedrine, or cocaine use, etc.

However, it remains undetermined if AMN—which afflicts eyes of predominantly young and female patients—portends any increased systemic cardiovascular risk, especially given that many conditions that prompt the development of AMN appear to be acute and one-off events. Elucidating this relationship is critical given the often young (and otherwise healthy) nature of AMN patients.

### 3.3. Retinal Imaging Biomarkers of Potential Cardiovascular Significance

#### 3.3.1. Paracentral Acute Middle Maculopathy

**Figure 4:**
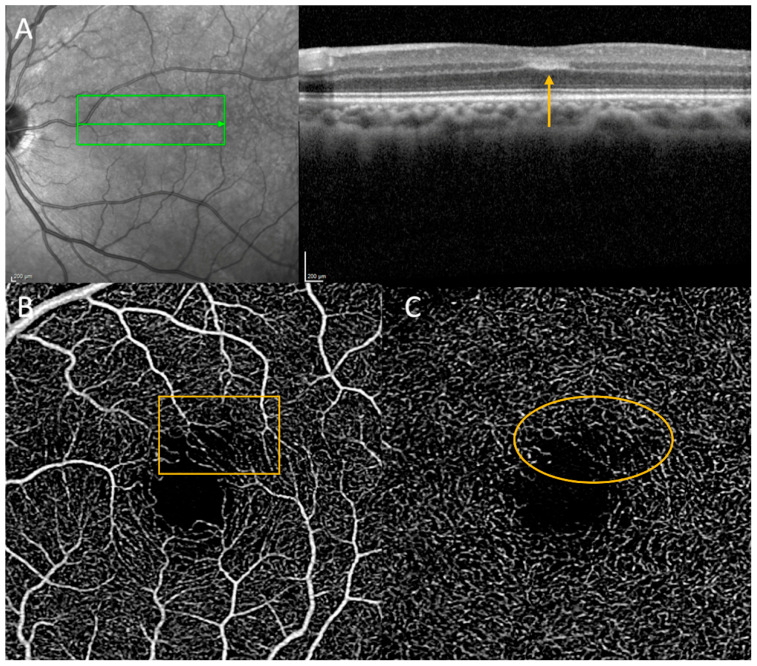
(**A**) Ocular coherence tomography demonstrating hyperreflectivity perifoveally in the inner nuclear layer consistent with paracentral acute middle maculopathy. (**B**) Ocular coherence tomography angiography demonstrating decreased flow signal superior and superotemporal to the fovea in the superficial vascular plexus corresponding to the PAMM lesion in Figure 4A (box). (**C**) Ocular coherence tomography angiography demonstrating decreased flow signal in the deep capillary plexus corresponding to the PAMM lesion in Figure 4A (oval).

Paracentral acute middle maculopathy (previously considered a variant of acute macular neuroretinopathy) is an OCT finding defined by the presence of a hyperreflective band at the level of the inner nuclear layer (Figure 4). It is presumed to indicate severe localized ischemia of the inner nuclear layer due to reduced flow of the deep and possibly intermediate retinal capillaries [61,62]. Patients often present with scotomas and subtle gray lesions on examination (duller and less opaque than cotton wool spots). Like CWS, PAMM lesions may be only transiently identified on fundoscopic exam; however, hyperreflectivity of the inner nuclear layer (without overlying outer retinal pathology or disorganization) is still detectable on OCT in the subacute stage. The overall incidence of PAMM is uncertain, and PAMM is sometimes considered an uncommon diagnosis. However, it may be present in asymptomatic patients, and PAMM lesions notably appear more frequently in eyes afflicted with forms of retinal vascular disease (e.g., RVO or RAO) or the fellow eyes of such patients [63].

As PAMM lesions are linked to dysfunctional flow in the retinal capillary plexuses, investigations into associated systemic cardiovascular pathology have been performed, with mixed results. Unsurprisingly, PAMM lesions are linked with other retinal vascular disorders such as CRAO, CRVO, or stigmata of hypertensive disease, all of which confer significant cardiovascular (stroke and myocardial infarction) morbidity risk [64]. PAMM lesions associated with ocular vaso-occlusive disease such as BRAO can herald critical carotid disease [65]. They also can be seen after rarer causes of retinal vascular ischemia with varying ramifications for systemic cardiovascular risk (e.g., Purtscher retinopathy and vasculitis of various etiologies, especially giant cell arteritis) [66,67]. There have been isolated case reports of PAMM heralding systemic cardiovascular disease, such as ischemic cardiomyopathy [68]. Yet isolated PAMM lesions without accompanying ocular pathology may not portend increased cardiovascular risk. A recent abstract from Moorfield’s Eye Hospital found no evidence that the presence of PAMM lesions confers significant added stroke or myocardial infarction risk in patients over 10 years of follow up. With only 47 patients enrolled, the study was likely underpowered [69]. However, a burgeoning body of thought suggests that a recently characterized retinal OCT finding, the retinal ischemic perivascular lesion (*RIPL*—discussed later in this article), may represent the legacy on OCT imaging of acute PAMM lesions. An increasing number of identified RIPLs on OCT appears to be linked to increased probability of systemic cardiovascular disease [70]. Thus, it is possible that increasing PAMM lesions, ultimately perceived as RIPLs by keen scanning of the macular cube, may indeed have prognostic significance on their own even in asymptomatic patients (see below).

Further research will be helpful in elucidating the progression of PAMM lesions temporally, especially on OCT. If future investigation establishes PAMM lesions as precursors to RIPLs, a careful cardiovascular history and potential referral and targeted workup (as described in the discussion on RIPLs) may be warranted. Currently, no systemic workup is recommended for isolated PAMM lesions.

#### 3.3.2. Retinal Ischemic Perivascular Lesions (RIPLs)

**Figure 5:**
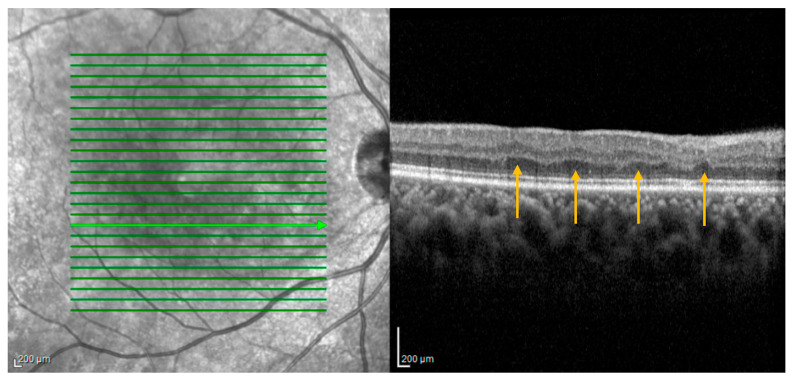
Ocular coherence tomography demonstrating several areas of focal atrophy of the inner nuclear layer (INL) with underlying expansion of the outer nuclear layer consistent with retinal ischemic perivascular lesions (RIPLs) (arrows).

Optical coherence tomography findings known as retinal ischemic perivascular lesions (RIPLs) were recently described in the literature [70]. RIPLs are characterized by focal atrophy of the inner nuclear layer (INL) accompanied by secondary expansion of the outer nuclear layer, resulting in an undulating appearance of the middle retinal layers (Figure 5). RIPLs are thought to arise at the sites of previous middle retinal layer infarcts, potentially evolving as a subsequent manifestation of paracentral middle maculopathy (PAMM), wherein the hyperreflective inner nuclear band observed during the acute phase gradually undergoes atrophy over time. PAMM has not been conclusively shown to confer increased cardiovascular risk. However, RIPLs do appear to be representative of end-organ infarction secondary to vascular dysfunction and are linked with cardiovascular disease.

A retrospective study of 160 subjects demonstrated a higher number of RIPLs in individuals with cardiovascular disease and, crucially, found that each additional RIPL identified on imaging conferred greater odds of cardiovascular disease, after adjusting for age, sex, and smoking status [70]. The presence of 1, 2, or 3 RIPLs was associated with an increased odds ratio of developing CVD of 2.3, 4.2, and 5.3, respectively, after adjusting for multiple covariables. Moreover, patients with intermediate or high 10-year ASCVD (atherosclerotic cardiovascular disease) risk scores were found to have more RIPLs than patients with low or borderline ASCVD risk scores. RIPLs have also been explored as an occult imaging biomarker for systemic cardiovascular disease. In a recent study, 8 of 11 (72.7%) patients with RIPLs and no prior diagnosis of cardiovascular disease were found to have newly diagnosed CVD upon further workup with their primary care physician or cardiologist [71]. Two of these patients required surgical intervention, including coronary artery bypass graft and carotid artery stent placement, and 3 were started on new medications. Despite the report’s limited sample size, the profound implications of the findings underscore the importance of considering cardiovascular evaluation in patients with RIPLs. These lesions, reasonably considered a new ophthalmic imaging biomarker of cardiovascular disease, may encourage ophthalmologists to refer their patients for age-appropriate cardiovascular disease screening (at minimum, serial blood pressure monitoring, lipid profile, and hemoglobin A1C). Patients in the aforementioned study also underwent electrocardiography and echocardiogram, with carotid duplex and ambulatory ECG also revealing abnormalities, in some cases resulting in invasive cardiac testing or surgical intervention.

Importantly, additional research is necessary to provide insight into the cost-effectiveness of referring patients with RIPLs for cardiovascular workup. Specifically determining which individuals would benefit the most from such evaluations and identifying standardized diagnostic tests to be included in the workup are viable future research goals with implications for personalized patient therapy.

### 3.4. Advanced Retinal Imaging Techniques and Correlations with Cardiovascular Disease

#### 3.4.1. Vessel Parameters on Fundus Photography

Fundus photography, a long-employed and basic ophthalmic imaging technique, can exhibit biomarkers of cardiovascular disease. Analysis of data from the Atherosclerosis Risk in Communities Study suggests that quantitative vessel diameter as measured by the artery-to-vein ratio correlates with systemic cardiovascular dysfunction. Risk of stroke and death increases as venular diameter increases and arteriolar diameter decreases [72]. Retinal microvascular changes as captured and measured on fundus photos may also reflect distant-site cardiovascular functioning; narrowing of small arterioles specifically seems to be associated with prior blood pressure levels 3 and 6 years prior to imaging [73]. Now, newer image analysis software and artificial intelligence have provided insight into retinal vascular phenotypes with increased CVD risk or concurrent cardiometabolic pathology. For instance, a study of the fundus photographs of 1069 patients with the Singapore “I” Vessel Assessment (SIVA) software categorized retinal vascular morphology and distribution and found three predominant patterns. One pattern (the “sparse vascular network”) explained a significant amount of variance (17.4%) in subjects’ cardiovascular disease risk (as measured by HEART score) [74]. Interestingly, a recent AI model used machine learning to predict myocardial infarction risk with minimal patient information. This model, when restricted solely to fundus photographs and demographic data of over 11,000 patients, could accurately estimate left ventricular mass, left ventricular end-diastolic volume, and crucially could predict the risk of myocardial infarction (AUC 0.80, sensitivity and specificity both >70%) [75]. These studies and those of similar aim and scope suggest that emerging software and artificial intelligence techniques show promise for clinical CVD risk stratification even on simple fundus photos.

#### 3.4.2. Retinal and Choroidal Thickness on Optical Coherence Tomography

Retinal and choroidal thickness on OCT have also frequently been studied as potential biomarkers of various cardiometabolic disease states and CVD risk. The measurement of the thicknesses of the ganglion cell and inner plexiform layer (GCIPL) and retinal nerve fiber layer (RNFL) have been extensively studied in many diseases such as glaucoma and have been found to be highly reproducible in healthy and sick eyes [76,77,78]. Choroidal thickness also appears to be a reproducible and repeatable measurement as well, though less studied [79,80].

The thickness of different layers of the retina, especially the GCIPL, have been associated with increasing adiposity (as measured by body mass index (BMI)). Increasing BMI appears significantly associated with thinning of the GCIPL, and an elevated BMI at baseline may be associated with decreased GCIPL over years of follow-up (8 years, in the case of this study) [81]. In fact, cardiovascular risk factors (such as lipid profile, blood pressure, tobacco smoking, and percentage of glycated hemoglobin) explained 12.6% of the variance in GCIPL thickness among subjects in the aforementioned study. Another recent study examined OCT-determined retinal nerve fiber layer (RNFL) thickness and CVD incidence. Across 25,563 participants from the UK Biobank without cardiovascular disease, 5% developed cardiovascular events (coronary heart disease, heart failure, stroke, or CVD-related mortality), and every 5 µm decrease in RNFL thickness was associated with an 8% increase in the development of cardiovascular events [82]. For comparison, the average age-associated decrease in RNFL thickness is approximately 0.3 µm per year [83]. Importantly, patients who reported a history of glaucoma were excluded, as the RNFL thinning incurred in glaucomatous optic neuropathy would preclude the utility of this imaging modality to assess CV risk.

The choroid also displays OCT imaging signatures that confer elevated CVD odds and risk. Age is a dominant influence on choroidal thickness as measured on OCT, and some studies have found no relationship between CVD and choroidal thickness after adjusting for age [84,85]. However, when patients (*N* = 193) with baseline subclinical atherosclerosis were divided into tertiles based on CVD risk (as determined by degree of coronary artery calcification on cardiac computed tomography), increasing coronary artery calcification was significantly associated with decreasing subfoveal choroidal thickness [86]. Other studies have reproduced these findings; patients with coronary artery disease have been found to have significantly lower subfoveal choroidal thickness on OCT (252 µm vs. 303 µm, *p* < 0.005) and also lower choroidal thickness throughout the fundus, especially the inferior macula [87]. Patients with acute MI also appear to have thinner choroids than controls, and patients with triple-vessel disease (but no acute MI) may have even thinner choroidal thickness on OCT scans relative to age-matched controls [88].

Notably, it is difficult to make clinically actionable strategies on these findings as choroidal thickness decreases with age and also decreases with increasing axial length of the eye. Additionally, while attempts have been made to create normalized data across various ages, there are currently no databases of reference that serve as standard of care [89].

#### 3.4.3. Optical Coherence Tomography Angiography

Optical Coherence Tomography Angiography (OCTA) has revolutionized the visualization of the retinal and choroidal vasculature. Specific information gathered by OCTA to characterize the structure and function of the retinal microvasculature includes vessel density (VD), vessel length, and perfusion density in the superficial, intermediate, and deep capillary layers of the retina and the choriocapillaris. The vessel density and size, perimeter, and topology of the fovea avascular zone (FAZ) are also readily resolved with OCTA [90]. Many studies have shown aberrations in these parameters in patients with various cardiovascular disease risk factors. OCTA thus has emerged as an important mode of imaging in evaluating both vascular retinal diseases and the burden of systemic cardiovascular dysfunction. OCTA quantification of different vascular parameters has also been found to be highly reproducible between different machine models and different operators, although the reproducibility and repeatability can vary with scan location and may be less than retinal and choroidal thickness measurements made by OCT [91,92,93].

OCTA of the retina has shown great promise as an imaging modality in assessing the systemic manifestations of hypertension and diabetes. The FAZ is enlarged on OCTA in patients with newly diagnosed hypertension and treated hypertension compared to healthy controls [94]. Vessel density in the deep capillary plexus and superficial capillary plexus has also repeatedly been found to be reduced in patients with hypertension, especially in the macula [95,96,97]. Indeed, even when previously diagnosed hypertensive patients were stratified into low-, medium-, and high-CVD-risk groups (based on presence of end-organ damage, blood glucose, lipid profile, and BMI, among other parameters), higher CVD risk significantly correlated with reduced perfusion density in the superficial and deep capillary plexuses in the macula [98]. Although this study did not find prominent pathology in the choriocapillaris, another recent study found increased choriocapillaris flow voids (more numerous flow voids, each of a smaller area) in hypertensive patients. Interestingly, 24-h blood pressure readings and the presence of diabetes also increased the strength of these findings [99]. These increased choriocapillaris flow voids may serve as a biomarker of systemic cardiovascular disease in undiagnosed patients in the future, especially when combined with characteristic decreases in VD and perfusion density in the superficial and deep retinal capillary plexus.

**Figure 6:**
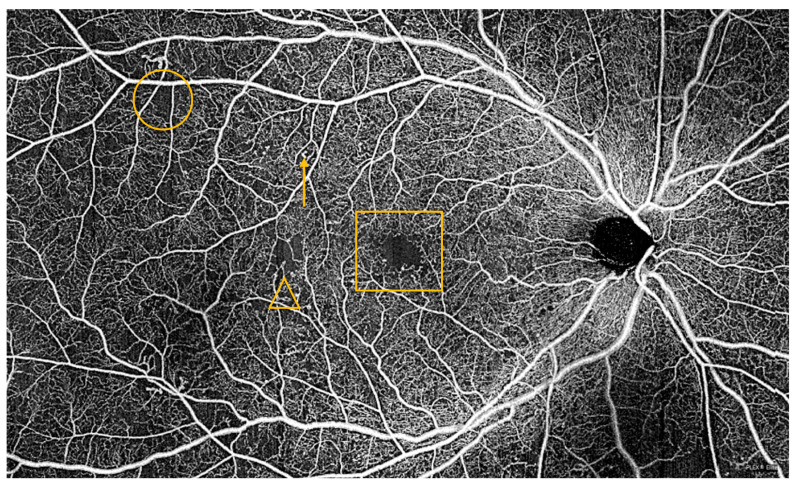

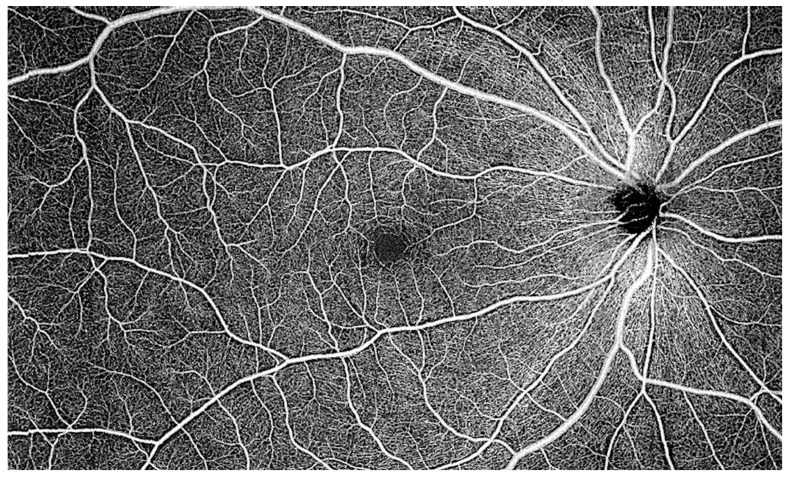
(**TOP**): Widefield ocular coherence tomography angiography of a patient with type 2 diabetes demonstrating an enlarged foveal avascular zone (box), microaneurysms (one of many, arrow), capillary nonperfusion (one area of many, circle), and venous looping (one of many, inside triangle). (**BOTTOM)**: Normal retinal vasculature as demonstrated on widefield ocular coherence tomography angiography as a comparison.

Additionally, retinas with cotton wool spots—a frequent finding in hypertensive and diabetic patients, as described in a previous section—unsurprisingly show significantly lower vessel density in the superficial capillary plexus around the lesion, and lower vessel density correlates with CWS area [100]. Vascular changes due to diabetes are also readily seen on OCTA. Increased foveal avascular zone area and decreased retinal capillary plexus vessel density are characteristic and can be monitored over time to chart the progression of vascular dysfunction secondary to diabetes (Figure 6) [101,102]. Decreasing VD, especially in the deep capillary plexus, correlates with the degree of diabetic retinopathy and vision impairment [103,104]. Furthermore, increasing FAZ and decreasing flow density significantly increase clinical prediction score accuracy of diabetic retinopathy progression when combined with traditional CVD risk factors, and decreasing vessel density of the superficial capillary plexus portends increased DME risk [105]. Indeed, these OCTA imaging biomarkers may hold predictive potential for CVD diagnosis and risk in diabetics. One prospective study of Black patients with diabetes found that heart disease, defined as prior diagnosis of heart failure, coronary artery disease, or myocardial infarction, significantly correlated with deep capillary plexus vessel density after multivariate analysis [106]. This provides further evidence in favor of this emerging, though nonspecific, imaging biomarker as reflective of general cardiovascular health.

OCTA findings have also been investigated as biomarkers for acute stroke and cardiac structure and dysfunction. Vessel density again has been identified as a possible imaging biomarker in acute ischemic stroke. Macular vessel density in the superior and deep capillary plexuses may be reduced in acute stroke patients [107]. Moreover, the degree of carotid artery stenosis, a critical stroke risk factor, correlates with decreasing superficial capillary plexus vessel density (but not deep capillary plexus VD) [108]. According to a recent *Journal of the American Heart Association* article, decreased superficial capillary vessel density is also correlated with higher left ventricular mass, interstitial volume, global longitudinal heart strain, and myocardial replacement fibrosis on cardiac magnetic resonance [109]. Superficial capillary plexus density is also associated with increased cardiovascular risk (using American Heart Association Risk and Global Registry of Acute Coronary Events scoring) and reduced left ventricular ejection fraction [110]. In fact, different sites of coronary artery stenosis (e.g., left anterior descending artery, right coronary artery) have variable associations with reduced vessel density of different retinal and choroidal capillary layers, but all were correlated with decreased fundus microvascular vessel density on OCTA [111].

Building on the literature examining retinal OCTA and cardiovascular disease, research is underway into machine learning (ML) applications and OCTA for the identification of cardiometabolic disease and risk. A recent study in *Eye* investigating two artificial intelligence models employing ML found that 3 × 3 mm OCTA retinal scans could be used to reliably predict the presence of hyperlipidemia, and also showed promise in identifying diabetes mellitus, hypertension, and congestive heart failure (AUC of receiver operator curve and accuracy > 0.5 for all) [112]. Retinal microvascular density analysis has also been utilized by machine learning models to identify controls versus acute stroke patients, with impressive results (AUC ranging from 0.66 to 0.81 with self-supervising deep learning intelligence prototypes) [113]. These machine learning analyses rely on findings imperceptible to human examiners and process them at scale and with rapidity. However, despite the exciting nature of this burgeoning field of research, intense future investigation is needed to validate these models. Moreover, among the select few common biomarkers most studied (e.g., vessel density and FAZ area), there are large overlaps in abnormal parameters seen in multiple states of increased cardiovascular risk or current disease.

## 4. Limitations

It must be noted that many of the imaging biomarkers discussed above are not specific to various forms of cardiovascular disease. For instance, many forms of dementia, such as Alzheimer’s and Parkinson’s Disease, have been associated with reduced vessel density in various layers of the retina when examined with OCTA [114,115,116]. Furthermore, parameters such as reduced vessel density in the various vascular layers of the retina are associated with multiple disease states of interest, such as chronic CVD (including hypertension and diabetes) and also acute stroke or MI risk. Therefore these biomarkers cannot “fingerprint” any one condition and cannot be used for individualized risk assessment as of yet. Despite the conclusions and investigations of the reviewed literature, the use of OCTA and OCT along with fundus photography to risk-stratify patients with cardiovascular disease or direct specific referral or treatment patterns is not yet indicated. These imaging biomarkers, though promising, remain exploratory in nature, and extensive future research and validation is required before they are implemented into routine screening paradigms.

This review article also has important limitations. Selection bias is a risk in any non-systematic review process such as this narrative review. Although best efforts were taken to prioritize original research over review articles and to evaluate each article on the merits of the data and conclusions presented, inclusion bias is possible. Additionally, literature of potential significance to the article was not included if it was not written originally in English or published with an accompanying translation.

## 5. Conclusions

Many retinal conditions and findings, both common and uncommon, herald future or current cardiovascular risk and should be considered when crafting individualized plans of care for patients. CRAO confers drastically elevated risk for acute stroke and myocardial infarction and chronic cardiac dysfunction such as atrial fibrillation. CRVO similarly has been shown to elevate stroke and MI risk (and statin therapy may ameliorate some level of risk, though significant future research is needed). Cotton wool spots are linked to significantly higher future risk of stroke- and heart-failure-related events, which is unsurprising given their tight association with diabetes and hypertension. Additionally, new research suggests that macular degeneration patients with SDD are at an increased risk of significant cardiac and vascular comorbidities—especially ischemic stroke and MI. However, despite the pathogenesis of PAMM and AMN OCT and OCTA findings involving ischemia at the level of the deep capillary plexus, neither have been definitively shown in the literature to confer elevated CVD risk, although PAMM may develop into RIPLs. The relationship between AMN and systemic CVD is likely understudied.

Different imaging biomarkers of the retina have also been evaluated for their relationship with cardiovascular disease. Decreasing artery-to-vessel ratio on fundus photography and decreasing retinal and choroidal thicknesses on OCT serve as biomarkers of systemic cardiovascular dysfunction. RIPLs, a novel OCT finding thought to arise at the sites of previous middle retinal layer infarcts, seem to be associated with the ensuing development of cardiovascular disease, or concurrent CVD (including structural pathology such as carotid stenosis requiring intervention).

Furthermore, OCTA is an emerging imaging modality, and multiple parameters such as the vessel density in the different capillary plexuses of the retina correlate with cardiovascular risk. These parameters may aid in personalized prognostication, workup, and treatment of CVD. However, these imaging biomarkers are nonspecific, and are also seen in other disease states such as various forms of neurocognitive dementia. Significant continued research and validation is required before they can be safely incorporated into clinical practice for screening and decision-making on further workup and treatment.

## Abbreviations

The following words and phrases are frequently abbreviated in this article: cardiovascular disease (CVD); central retinal artery occlusion (CRAO); retinal artery occlusion (RAO); retinal vein occlusion (RVO); central venous artery occlusion (CRVO); transient ischemic attack (TIA); myocardial infarction (MI); coronary artery disease (CAD); cotton wool spots (CWS); age-related macular degeneration (AMD); subretinal drusenoid deposits (SDD); paracentral acute middle maculopathy (PAMM); acute macular neuroretinopathy (AMN); retinal ischemic perivascular lesion (RIPL); inner nuclear layer (INL); optical coherence tomography (OCT); optical coherence tomography angiography (OCTA); retinal nerve fiber layer (RNFL); ganglion cell and inner plexiform layer (GCIPL); foveal avascular zone (FAZ); and vessel density (VD).
